# Experiences and Expectations for Glioma Immunotherapeutic Approaches

**DOI:** 10.3389/fonc.2014.00355

**Published:** 2014-12-10

**Authors:** Ryuya Yamanaka, Azusa Hayano

**Affiliations:** ^1^Graduate School for Health Care Science, Kyoto Prefectural University of Medicine, Kyoto, Japan

**Keywords:** central nervous system, CTLA4, dendritic cell, genetically engineering T cells, glioma, immunotherapy, PD-1, peptide

Malignant gliomas are the most prevalent type of primary central nervous system (CNS) tumor in adults. Despite progress in brain tumor therapy, the prognosis for malignant glioma patients remains dismal. Standard treatment with temozolomide and radiotherapy for patients with newly diagnosed glioblastoma has increased the median overall survival (OS) by 15–20 months (1), but tumor recurrence is inevitable. Salvage treatments upon recurrence are palliative at best and rarely provide significant survival benefit. Among the new treatments currently being investigated for malignant glioma, immunotherapy is theoretically attractive, because it offers the potential for high tumor-specific cytotoxicity (2). Although recent clinical trials of immunotherapy protocols for malignant gliomas focused on initiating and amplifying a host response with some clinical success, most of them failed to induce objective tumor shrinkage in patients (2). Antitumor activities of tumor cytotoxic T cells (CTL) and antibodies induced by these therapies are insufficient to overcome tumor growth because tumors have immune evasion mechanisms instigated by myeloid derived suppressor cells (MDSCs) and regulatory T cells (T_reg_) (3). In this paper, we will review past experiences and discuss the promising future of immunotherapeutic approach for glioma treatment.

## What have We Learned from Previous Clinical Trials?

Preliminary results from recent immunotherapeutic clinical trials (2, 4–6) with dendritic cells or peptide vaccines for malignant glioma patients are encouraging. However, these trials have some limitations, and we will have to await the results of several phase III trials to make definitive conclusions. There are several concerns from past experiences.

1. The immune responses such as CTL and antibody production were not sufficient to overcome glioma progression, and were not correlated to clinical outcomes.
2. New issues have emerged regarding the evaluation of disease response, and with the identification of patterns such as pseudoprogression (7) that is frequently indistinguishable from disease progression. Additionally, there are delayed radiation responses after radiotherapy. In short, there are pitfalls in distinguishing the response of radiotherapy to that of immunotherapy.
3. There are prognostic variations and long term survivors among glioblastoma patients (8). We therefore have to develop molecular markers to predict the prognosis of the patient more precisely to conduct clinical trials with less bias.
4. We have to develop biomarkers that predict patients’ responses to individualized immunotherapy. To do so, we have to conduct clinical trials that exclude patients with pseudoprogression, a delayed radiation response and a biologically good prognostic group.
5. Most immunotherapy clinical trials state that the therapy is safe. This is a concern because the adverse events of immunotherapy are usually interpreted as those of the clinical course of glioma. We have to continue to carefully monitor patients, because acute disseminated encephalomyelitis and neuropathic syndrome following vaccination against human papillomavirus for cervical cancer are now serious problems (9).
6. In recent years, there has been a significant increase in OS and progression free survival (PFS) owing to improvements in standard of care (10). In phase II clinical trials, survival data are usually compared to that of a decade ago, so emerging therapeutics are easily misconstrued as effective therapies.
7. In Japan, bevacizumab was approved for glioblastoma in June 2013. Therefore, we should reconsider whether an immunotherapeutic approach for glioma could be a new standard of care.
8. In Japan, medical oncologists are expected to participate in the development of global immunotherapeutic protocols for glioblastoma.

## Prognostic Markers for Glioblastoma

The World Health Organization (WHO) currently has the most widely used system for prognostic markers; a high WHO grade correlates with clinical progression and decreased survival rate (11). However, individual fates vary within diagnostic categories. There are several prognostic factors that are associated with longer survival of glioblastoma patients, including age, performance status (PS), *MGMT* status, and *IDH1* mutation. The inadequacy of histopathological grading is shown, in part, by the inability to recognize patients prospectively. We and other researchers have developed a predictive method for patient outcome that enables clinicians to make optimal clinical decisions using microarray technology (8, 12–14). Our work described an expression profiling study of glioblastoma patients for the identification of genes that predict OS using random survival forests models (8). The gene expression predictor, which we named the Prognosis Prediction Score (PPS), was computed from a linear combination of 25 selected genes and was calculated for each tumor as follows:

$$\begin{array}{llll} Z_{1} & =0.\mathrm{27}\times \mathrm{GPNMB}+0.09\times \mathrm{EFNB}2\; & & \;\\ & \;-0.\mathrm{22}\times \mathrm{ASF}1A+0.02\; & & \;\\ & \;\times \mathrm{LOC}283027+0.\mathrm{15}\times \mathrm{AMIGO}2\; & & \;\\ & \;+0.\mathrm{22}\times \mathrm{IL}13\mathrm{RA}2+0.\mathrm{25}\times \mathrm{ITGA}7\; & & \;\\ & \;+0.\mathrm{15}\times \mathrm{LDHA}-0.01\times C11\mathrm{orf}71\; & & \;\\ & \;+0.\mathrm{15}\times \mathrm{AFTPH}+0.\mathrm{15}\; & & \;\\ & \;\times \mathrm{TBC}1D19-0.\mathrm{21}\times \mathrm{MED}29\; & & \;\\ & \;+0.02\times \mathrm{ACN}9+0.\mathrm{29}\; & & \;\\ & \;\times \mathrm{SLC}25A19+0.\mathrm{16}\times \mathrm{RPL}12\; & & \;\\ & \;-0.09\times \mathrm{ALS}2\mathrm{CR}4-0.\mathrm{14}\; & & \;\\ & \;\times C10\mathrm{orf}88-0.\mathrm{11}\times \mathrm{ARHGAP}39\; & & \;\\ & \;+0.\mathrm{18}\times \mathrm{LMAN}2L+0.\mathrm{29}\times \mathrm{CASP}8\; & & \;\\ & \;-0.\mathrm{28}\times \mathrm{ST}6\mathrm{GAL}2+0.\mathrm{33}\times \mathrm{LOXL}3\; & & \;\\ & \;+0.08\times \mathrm{ANGPTL}1+0.\mathrm{22}\times \mathrm{MRRF}\; & & \;\\ & \;-0.\mathrm{33}\times \mathrm{ARHGAP}32.\; & & \; \end{array}$$

**Figure 1:**
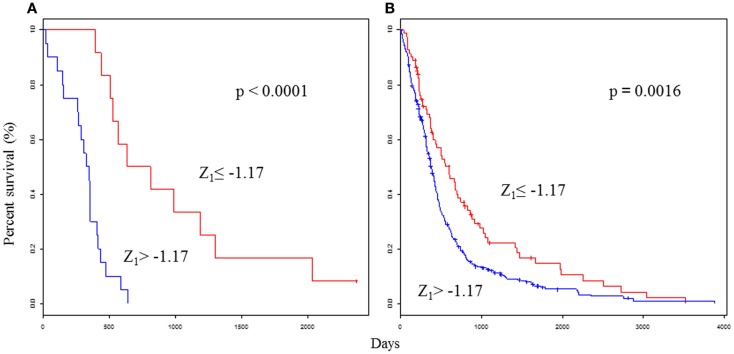
**Survival analyses using the selected 25 gene classifiers show prognostic value for glioblastoma**. Kaplan–Meier curves that compare groups classified by the Z_1_ PPS with the 25 gene model in the test set **(A)** and validation set **(B)**. Permission for reuse was obtained from John Wiley & Sons Ltd. and ©2013 Japanese Cancer Association.

As expected, the predictor performed well in terms of patient prognosis: the improved prognosis group (Z_1_ ≤ −1.17) had a median survival time of 721 days, while the poor prognosis group (Z_1_ > −1.17) had a significantly lower median survival time of 335 days (*P* < 0.0001; Figure 1A). For more practical purposes, the PPS could also be computed from a linear combination of three genes and was calculated for each tumor as follows:

$$Z_{2}=-0.63\times \mathrm{ASF}1A+0.62\times \mathrm{ITGA}7+0.47\times \mathrm{AFTPH}.$$

As expected, the predictor performed well in terms of patient prognosis: the improved prognosis group (Z_2_ ≤ −0.76) and the poor prognosis group (Z_2_ > −0.76) had identical median survival times and significance scores as Z_1_. The Z PPS results were compared with traditional individual indicators. Z_1_, Z_2_, age, PS, and subtype were significantly associated with OS in univariate analyses. Z_1_ was significantly associated with OS by multivariate analyses. The PPS was the most significant feature of these clinical parameters. The PPS formula was validated in the validation set (*n* = 488), which was derived from glioblastoma patients in four external data sets (12–15). As expected, the OS was significantly higher in the improved prognosis group (Z_1_ ≤ −1.17) than in the poor prognosis group (Z_1_ > −1.17) (*P* = 0.0016; Figure 1B). Two-year survival rates were 36.3 and 30.8% in the improved prognosis group, and 4.7 and 11.8% in the poor prognosis group, using the test and validation data sets, respectively. Even among glioblastomas in both test (*n* = 32) and validation sets (*n* = 488), the OS ranged between 0 and 3,880 days. Fifty-two patients (10%) survived for longer than 1,000 days. Class prediction models based on defined molecular profiles allow the classification of malignant gliomas in a manner that will better correlate with clinical outcomes than with standard pathology. Glioblastomas have a wide-ranging survival time, which requires a more precise prognostic scoring system to study novel therapeutic approaches. Therefore, the identification of molecular subclasses could greatly facilitate our ability to develop effective treatment protocols.

## Future Perspectives

The genetic landscape of gliomas has been revealed by the advancements of genome sequencing technology (15, 16). Researchers are now trying to develop novel therapeutic strategies based on these exciting discoveries. New therapeutic strategies, such as targeted therapies and anti-angiogenic treatments that appear promising with regard to improving the results have been reported (17). Immunotherapies have also shown promise for treating advanced solid tumors. In particular, monoclonal antibodies that block inhibitory immune checkpoint molecules and enhance the immune response to tumors such as cytotoxic T-lymphocyte-associated antigen 4 (CTLA4) and programed cell death protein 1 (PD-1) (18, 19). Another forefront of immunotherapy research is genetically engineering T cells to target tumor cells (20). Future efforts will need to focus on development of novel therapies that appear active as monotherapies or in combinatorial regimens that modulate the host immune system. Although it is still unknown whether these novel discoveries will be suited for use in the CNS microenvironment, we are awaiting the next generation of progress for glioma immunotherapy based on the fundamental pathophysiology of this challenging disease.

## Conflict of Interest Statement

The authors declare that the research was conducted in the absence of any commercial or financial relationships that could be construed as a potential conflict of interest.
